# Do Former Elite Athletes Live Longer? New Evidence From German Olympic Athletes and a First Model Description

**DOI:** 10.3389/fspor.2020.588204

**Published:** 2020-11-06

**Authors:** Lutz Thieme, Michael Fröhlich

**Affiliations:** ^1^University of Applied Science Koblenz, Koblenz, Germany; ^2^Technische Universität Kaiserslautern, Kaiserslautern, Germany

**Keywords:** mortality, longevity, elite athletes, survival, Olympian

## Abstract

The positive effects of physical activity and recreational sports on health have been well-examined and are well-proven. In contrast, the consequences of extensive elite sports on life expectancy and mortality rates have been described in significantly less unique and comprehensive terms. There is a lack of models that systematically summarize the factors influencing the life span of elite athletes. Therefore, this study identifies the difference between all 6,066 German participants in Olympic Games between 1956 and 2016 and the total population, as well as between participants from the Federal Republic of Germany (FRG) and the German Democratic Republic (GDR), and between the genders. Currently, the survival rate of German Olympians is lower compared to the general population. On the contrary, it was found that Olympic success represents a linear risk for survival probability. While different types of sports do not exhibit any differences, gender and origin (FRG vs. GDR) do represent a significant risk factor. These results are combined with the current state of research to create an impact model of factors influencing the life span of elite athletes.

## Introduction

The sustained positive effects of physical activity and recreational sports with moderate to slightly intensive exertion on health, well-being, mobility, and mortality have been well-examined and empirically proven (Blair et al., 1995; Kujala et al., 1998; Garber et al., 2011; Turi-Lynch et al., 2019). In contrast, empirical evidence of long-term health effects caused by extensive elite sports and of the associated life expectancy is less comprehensive and more heterogeneous (Beaglehole and Stewart, 1983; DeKosky et al., 2018).

To estimate long-term health effects resulting from elite sports, survival time analyses are applied. These analyses compare survival rates of elite athletes for a specific country with those of the country's general population, or they examine the survival rates of elite athletes within various subpopulations (Lee-Heidenreich et al., 2017; Nguyen et al., 2019). Common comparison parameters include the standardized mortality ratio (SMR) (Bland, 2015) and survival curves or functions (Altman, 1999).

Looking at the mortality of elite athletes compared to the general population, the mortality rate of Olympians is consistently lower (Clarke et al., 2012; Coate and Sun, 2013; Antero-Jacquemin et al., 2014, 2015; Lin et al., 2016; Radonić et al., 2017; Keller, 2019). For example, Japanese Olympic athletes live longer than the overall Japanese population (Takeuchi et al., 2019). However, more frequent Olympic participation and a higher intensity of the sport practiced are associated with higher mortality.

Compared to the general population, lower mortality rates were also found in French Tour de France participants between 1947 and 2012 (Marijon et al., 2013), in 302 male and female finalists of the British and US American tennis championships since the 1880s (Coate and Sun, 2013), as well as in a group of 3,439 NFL players in the 1959–1988 seasons (Lehman et al., 2012).

Meta-analyses and systematic reviews by Garatachea et al. (2014), Lemez and Baker (2015), and Teramoto and Bungum (2010) consistently showed higher life expectancies or lower mortality rates in elite athletes vs. the comparative population, although Venkataramani et al. (2018) found no differences between 3812 National Football League players in the 1982 to 1992 seasons and the general population (Beaglehole and Stewart, 1983; DeKosky et al., 2018).

In their meta-analysis, Teramoto and Bungum (2010) identified primarily endurance sports with aerobic energy provision (e.g., long-distance running and cross-country skiing) and mixed sports with both aerobic and anaerobic energy provision (e.g., soccer, ice-hockey, basketball) as influencing factors leading to higher life expectancies and a lower mortality risk. The results by Ruiz et al. (2014) on marathon runners, professional cyclists, and Olympic athletes confirm these results. Moreover, team athletes seem to have higher survival rates than individually performing athletes.

A comprehensive comparison of African American, Hispanic, and Caucasian Major League baseball players, including comparisons of the respective ethnic groups from the years of birth between 1905 and 1966, was submitted by Markowitz (2019) without a clear finding in favor of team sports.

In a study that has been the only one to examine German elite athletes so far, Kuss et al. (2011) identified a slightly increased mortality rate in 812 soccer players between 1908 and 2006, as well as a life expectancy reduced by an average of 1.9 years.

In all studies, however, it is unclear whether the comparison group was the total population or the population from an age at which participation in the Olympics is usually possible. A comparison with the total population would include the higher infant mortality even though Olympic participants have already passed this stage of life.

This study adds the results of mortality analyses of all German Olympic participants from 1956 to 2016 to the international state of research. This is particularly interesting because we considered both the Federal Republic of Germany (FRG) and the German Democratic Republic (GDR), two German states with different social, economic, and sports systems which existed for more than 40 years and exhibited different developments in the life expectancy of the entire population. While Thieme (2020) focuses on the political aspects of this data, in this paper, we propose a first model. The model is based on the results of our analysis of the causes for the life span of elite athletes and builds on the current state of research. So far, such model has not been found in any relevant publication.

## Materials and Methods

### Data Collection

It proved to be impracticable to obtain death data from official German registers. The decentralized civil status register would require knowing the last place of residence of the deceased which is difficult to obtain for the large data set. In addition, the records have only been kept electronically since 2009. Instead, we used data from the largest and most reliable website on the participants of the Olympic Games (https://www.sports-reference.com/olympics.html, reopened under Olympedia.org since May 2020). In addition, two experts from large sports organizations provided references pertaining to deaths.

Randomly selected data points, which made up about 25% of the data set, as well as data points that appeared implausible were cross-checked against Wikipedia [e.g., for the West German participants in the 1972 Winter Olympics see https://de.wikipedia.org/wiki/Olympische_Winterspiele_1972/Teilnehmer_(BR_Deutschland)] and relevant chronicles of the Olympic Games. In case of discrepancies between two sources, further research was carried out, starting with the data sources cited in Wikipedia. The error rate of the cross-checked data points was below 3% and mainly concerned deviations in birth and death dates of a few days. Nevertheless, it is possible that some deaths were not recorded. However, we do not believe there to be any systematic distortions between the subgroups analyzed.

The survey period was limited to 1956–2016 because data on the deaths of Olympians in the first half of the 20th century was incomplete, and the impact of World Wars I and II could not be controlled in an adequate manner. Overall, 8,934 German Olympians were identified for the 60-year survey period. After eliminating duplicate athletes, 6,066 athletes remained, of which 4,023 participated in one, 1,436 in two, 442 in three, and 165 in four or more Olympic Games. Our data set of all German Olympic participants from 1956 to 2016 and their life spans are summarized in Table 1. In addition, the data set included first name, last name, date of birth, date of death, year(s) of Olympic Games participation, type of sports, number and types of medal wins, and origin of their team (FRG/GDR/reunified Germany).

**Table 1:** Description of the exposure group.

	**German Olympic participants 1956–2016**			**German Olympic participants 1992–2016**			**FRG Olympic participants 1956–1988**			**GDR Olympic participants 1956–1988**		
	**All**	**Female**	**Male**	**All**	**Female**	**Male**	**All**	**Female**	**Male**	**All**	**Female**	**Male**
*N*	6,066	1,959	4,107	2,325	981	1,344	2,107	478	1,629	1,634	500	1,134
**Deceased (D) and life span (LS)**												
*N*_D_	400	37	363	7	1	6	275	21	254	118	15	103
Share of de-ceased (%)	6.59	1.89	8.84	0.30	0.10	0.45	13.05	4.39	15.59	7.22	3.00	9.08
Min. LS (months)	234	234	293	234	234	317	282	282	293	341	584	341
Max. LS (months)	1,118	1,088	1,118	584	234	584	1,104	1,088	1,104	1,118	1,088	1,104
Mean LS (months)	788.0	740.6	792.8	405.3	234.0	433.8	803.7	770.3	806.4	774.1	757.7	780.1
Standard deviation (months)	183.1	222.7	178.3	129.7	–	115.6	178.6	244.3	172.5	171.0	120.1	173.6

### Statistical Analysis

For our analysis, we determined the standardized mortality ratio (SMR), describing the ratio of the actually observed number of deaths of Olympians to the expected number of deaths calculated based on the general population. An SMR above 1 indicates a higher number of deaths among Olympians than expected for the general population, and the higher the SMR, the larger the number of deaths compared to the expectation.

To calculate the SMRs, we classified each Olympic participant based on the year of their first participation and the origin of their team (FRG: 1956–1988, *n* = 2,107; GDR: 1956–1988, *n* = 1,634; reunified Germany: 1992–2016, *n* = 2,325).

We obtained mortality tables from the Human Mortality Database (https://www.mortality.org/) and, based on the mortality rates of the total population of the FRG, GDR, and reunified Germany for the respective age groups starting from the age of 15, calculated the cumulative probability of death. The resulting mortality risk was then used to calculate the expected deaths in the respective study groups.

Of the 6,066 German participants in Olympic Games between 1956 and 2016, 400 died by the end date of our study, July 1, 2019. Since the number of deaths among Olympians was not sufficient to determine the probability of death for each year group in each year of the observation period, we aggregated the data of both the observation group and the general population. Meaningful results were achieved by aggregating the data into three time ranges (death between 1956 and 1974, between 1975 and 1994, and between 1995 and 2017 or 2019, depending on the type of analysis) and, per time range, four age groups (0–14, 15–34, 35–64, 65 years and older at the time of death). Smaller time ranges and age groups could deliver additional evidence. The age group of 0–14 years was not taken into account because participants in the Olympic Games are typically above 14 years old. Moreover, taking into account the higher child mortality would distort the results, making the results appear more favorable for Olympic participants. Thus, the following results and discussions refer to the three age groups listed above and exclude the first one.

We analyzed three subgroups: FRG vs. GDR, female vs. male, and different types of sport. The Wilcoxon–Gehan significance test (Gehan, 1965; Bühl, 2019) was used to test for differences in the life spans of these subgroups. In addition, differences between the subgroups were calculated applying the Kaplan–Meier estimator (Gaus and Muche, 2014), and we checked for significant differences using the log-rank test. Cox regressions included forward and backward selection of variables (VanderWeele, 2019) and helped identifying the variables that affected the relative mortality risk (hazard ratio) and their degree of impact (Zwiener et al., 2011; Gaus and Muche, 2014). The critical event in the Cox regressions is death. The number of months of life until death, or the key date of the examination, is used as the dependent variable. The time of first-time participation in the Olympic Games is included in the models as a regressor and is used as a proxy for the general increase in life expectancy within the Cox regression. The procedures applied are standard procedures used in medical statistics for survival analyses (Altman, 1999; Klein and Moeschberger, 2003; Bland, 2015). *P* < 0.05 were considered to indicate statistical significance. Data analysis was conducted using IBM SPSS Statistics software version 25 (Chicago, IL, USA).

Our analysis strategy is based on the variables currently available. It does not yet allow for any causal conclusions, confounder analyses, or sensitivity analyses. It can only be a first step toward a causal analysis, disturbance variable analysis, and sensitivity analysis. The knowledge gained from the results of our analysis, in addition to the current state of research, will be used below to propose a model of impact factors on the life span of elite athletes.

## Results

### Comparison With the General Population in East and West Germany

The data in Table 1 shows the number of all German Olympic participants between 1956 and 2016 and the three sub-risk groups analyzed (German Olympic participants 1992–2016, FRG Olympic participants 1956–1988, GDR Olympic participants 1956–1988). For all groups, the total size, the number of women and men (row N), the respective number of deceases (row N_D_), and the percentage of deceases (row “Share of deceases”) as well as information on the maximum, minimum, average, and standard deviation of the months of life (LS) in the observation period are listed.

In order to compare the mortality risk of West (FRG) and East German (GDR) Olympic participants with the general population aged 15 and over, the mortality risks of the general population were aggregated across the observation periods (1956–1974; 1975–1994; 1995–2017) and age groups (15–34; 35–64; 65 and older) and multiplied by the number of associated groups of Olympic participants. The resulting expected number of deaths was compared to the observed number of deaths. The observed and expected number of deaths determined for the three time periods and the three age groups and the resulting SMRs are shown in Table 2.

**Table 2:** Age group- and time range-specific SMR differentiated by FRG and GDR.

**Time range/age group**	**West German Olympic team in relation to West German population**			**East German Olympic team in relation to East German population**		
	**Deaths observed**	**Expected deaths**	**SMR**	**Deaths observed**	**Expected deaths**	**SMR**
**1956–1974**						
15–34	10	7.48	1.34	0	5.43	–
35–64	5	8.04	0.62	2	3.49	0.57
65+	2	0.71	2.80	Not an exposure group		
**1975–1994**						
15–34	13	5.86	2.22	9	5.77	1.56
35–64	40	40.44	0.99	21	34.36	0.61
65+	18	12.84	1.40	2	2.49	0.80
**1995–2017**						
15–34	4	0.37	10.71	1	0.35	2.86
35–64	96	47.26	2.03	63	47.61	1.32
65+	271	239.57	1.13	103	152.29	0.68

Comparing West German Olympians, defined by members of the joint German Olympic teams between 1956 and 1964 who were nominated by West Germany, and/or members of the FRG team between 1968 and 1988, with the West German population, the age group of 15–34 shows an increased SMR for all three time ranges. Instead of the expected 7.48 deaths between 1956 and 1974, there were 10 deaths (SMR_w15−34;1956−1974_ = 1.34), 1975–1994 experienced 13 instead of the expected 5.86 deaths (SMR_w15−34;1975−1994_ = 2.22), and four instead of 0.37 deaths occurred between 1995 and 2017 (SMR_w15−34;1995−2017_ = 10.71), with two of the four deaths between 1995 and 2004 having occurred in the age group of 25–34 years old. Female athletes are more affected by the increased mortality risk of the 15–24-year-old West German Olympians between 1956 and 1974 and between 1995 and 2017 than male athletes.

In the age group of 35–64, the mortality risk between 1956 and 1974 and between 1975 and 1994 is below that of the general population (SMR_w35−64;1956−1974_ = 0.62; SMR_w35−64;1975−1994_ = 0.99). For the latest period, however, it is twice as high (SMR_w35−64;1996−2017_ = 2.03), which, however, applies to the male athletes only.

The mortality risk of athletes aged 65 years and above decreases over the three time ranges. The triple risk for the West German Olympians between 1956 and 1974 (SMR_w65+;1956−1974_ = 2.80) is halved (SMR_w65+;1975−1994_ = 1.40) and only slightly exceeds the mortality risk of the general population in the most recent time range (SMR_w65+;1995−2017_ = 1.13).

An increased mortality risk was also determined for the East German Olympians between 15 and 34 years old in comparison with the GDR population or the East German federal states (see Table 2). The higher mortality risk pertained mostly to male athletes but was below that of the Olympians from the West German federal states with SMR_o15−34;1975−1994_=1.56 and SMR_o15−34;1995−2017_ = 2.86.

Thirty-five- to sixty-four-year-old East German Olympians exhibit a significantly reduced mortality rate (SMR_o35−64;1956−1974_ = 0.57; SMR_o35−64;1975−1994_ = 0.61) in the first two time ranges when compared to the East German general population. While female athletes remain at a comparable level during the most recent time range (SMR_oF35−64;1995−2017_ = 0.60), the mortality risk for male Olympians is considerably higher than that of the overall male population (SMR_oM35−64;1995−2017_ = 1.32).

Due to the low number of deaths (*n* = 7) for Olympic participants from 1992 onward (*n* = 2,325), no reliable statement can yet be made about the youngest generation of German athletes. In purely mathematical terms, the SMR is slightly higher (SMR_G15−34, 95−17_ = 1.19).

### Standardized Mortality Ratio Development for German Olympians

**Figure 1:**
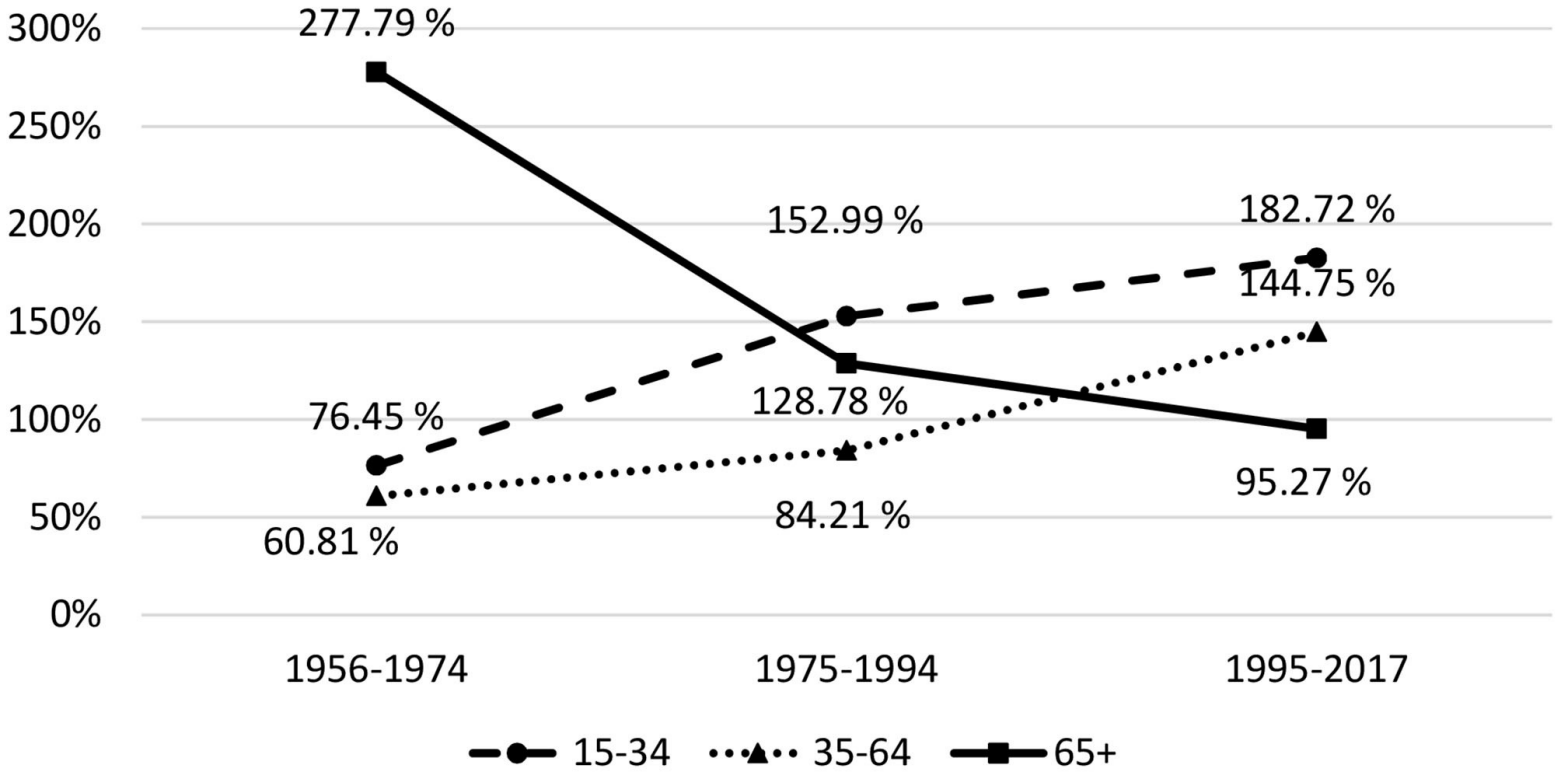
Development of the standardized mortality rates (SMR) by age group and time range.

For the three time ranges analyzed, the entire group of Olympians between 1956 and 2016 from East and West Germany shows higher standardized mortality rates in the age groups 15–34 and 35–64, and a lower mortality rate for athletes aged 65 and above (see Figure 1).

Analyzing the development of the SMR over time in age groups of 10 years each, starting from the age of 15, for the time ranges 1956–1964, 1965–1974, etc., up to 2005–2017, and taking into account the respective regression lines, it can be seen that the SMR increases for both age groups 15–24 and 25–34, and decreases for the six older age groups. This indicates that athletes' mortality risk for younger age groups develops negatively compared to the general population, while it converges toward the general population for older age groups.

### Differences Between Olympians From the FRG and the GDR

**Figure 2:**
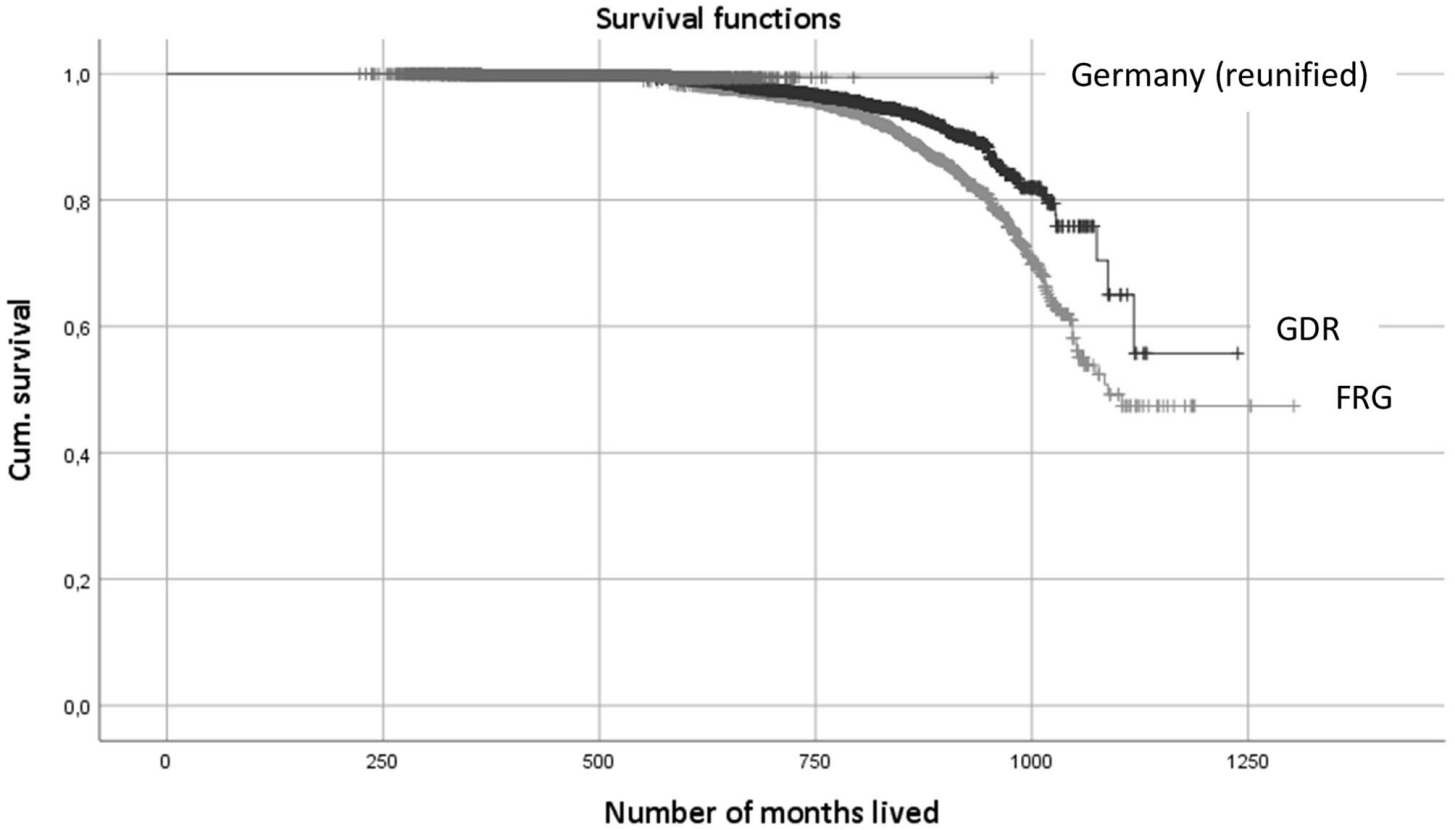
Kaplan-Meier curve for German Olympians (including censored Data).

Significant differences (*p* < 0.01) are identified when applying the Wilcoxon–Gehan statistics for comparing survival functions between the GDR Olympians (*n* = 1,634; 118 deaths) and the FRG Olympians (*n* = 2,107; 275 deaths), who participated in Olympic Games between 1956 and 1988 for the first time (see Figure 2). FRG athletes have a higher SMR (SMR_FRG_ = 1.24; CI: 1.10; 1.42) than GDR athletes (SMR_FRG_ = 0.69; CI: 0.61; 0.79). The same result is obtained when applying the log-rank test within the Kaplan–Meier method (*p* < 0.01).

### Differences Between Female and Male Olympians

The comparison between German female Olympic participants (*n* = 1,959, 37 deaths) and male Olympic participants (*n* = 4,107, 363 deaths) reveals a significantly higher probability of survival (*p* < 0.01) of female Olympic participants (SMR_Men_ = 1.34; SMR_Women_ = 0.29). This is also the case when separately comparing the genders in the teams of the FRG (*p* < 0.01) and the GDR (*p* < 0.05). Due to the low number of deaths of athletes participating in the 1992 Olympic Games or later for their first time, no conclusions can be drawn yet with respect to mortality rates of reunified Germany.

### Determining Factors of German Olympians' Life Expectancy

Using the Cox regression analysis, it appears that in addition to coming from the FRG (only in the model without the participants after 1988), being male represents the second most significant risk factor (HR_G_ = 2.18; CI: 1.54;3.09; *p* < 0.01) among the risk factors pertaining to survival probability. Further significant risk factors are the total number of medals won (HR_M_ = 1.22; CI: 1.08; 1.37; *p* < 0.001) and the year of first participation in the Olympic Games (HR_OT_ = 0.97; CI: 0.96; 0.987; *p* < 0.000) as a proxy for the increase in life expectancy over time. In summary: the better the Olympic result, the higher the mortality risk in this cohort. Non-significant factors within the Cox regression were the age at first Olympic participation, the differentiation between winter and summer games, the number of Olympic participations, and the differentiation between team, individual, and mixed sports. In a separate Cox regression, we also included prototypical aerobic and anaerobic sports as independent factors but did not detect any significant effects on life span. We obtained the same result when using doping-related sports as an additional explanatory factor.

## Discussion

An exposure group's mortality rates always show the sum of positive and negative factors effective at a specific measurement point in time in comparison to a control group (Kalwij, 2018; Nguyen et al., 2019). This means that the results are always preliminary. Nevertheless, since 1956, the sum of positive and negative effects of elite sports on the mortality of German Olympic participants appears to have been negative in comparison to the general population over 14 years old. What is surprising is that we did not find any evidence in our data set and analysis that assumed risk factors such as being part of the East German Olympic team or sports-specific risks indeed impact the mortality rate. The analysis by Nguyen et al. (2019) of professional American Football and American Baseball players and the analysis by Lee-Heidenreich et al. (2017) of high jumpers, marathon runners, and sprinters showed different mortality rates across the types of sports analyzed.

The lower mortality rates of elite athletes in most countries other than Germany compared to the respective country's general population (Gajewski and Poznańska, 2008; Kalwij, 2018) could possibly be explained by the comparatively high life expectancy in Germany, as well as by the lower socioeconomic profit generated from a career in elite sports in Germany. This is also indicated by the findings of Wicker et al. (2020), which show that German elite athletes generally have a lower life satisfaction compared to the overall population of the same age. Lower mortality rates of athletes with high metabolism contradict theories of aging (Prinzinger, 2005). In contrast, the here presented study shows a negative influence on life span when participating in the Olympic Games several times and thus developing a high metabolism for a longer time. It also appears to be the case that previous studies compared the mortality of elite athletes with the total population, including infants. The higher infant mortality may, however, make the results appear more favorable for Olympic participants.

Like all previous studies on the mortality of elite athletes, the discussion of the data used for our study is based on the assumption that elite sports influence the exposure group's life expectancy (Abel and Kruger, 2005; Gajewski and Poznańska, 2008). It is, of course, also possible that elite sports act as a selection factor: people whose genetic predisposition leads them to living a riskier life may be more likely to choose elite sports, so that they as a group have a different life expectancy than the general population. This would mean that elite sports represent a variant of different lifestyles in which people with specific dispositions prevail. The findings on the Goldman-Dilemma (Gonzalez et al., 2018) are, for example, an indication of the willingness of elite athletes to take risky decisions. We call this the endogeneity problem.

The findings from previous research and the newly presented findings on German Olympic participants can be combined into a research strategy that aims to causally explain the effects of extensive elite sports on mortality risk, and to isolate the effect factors. In mortality studies, socioeconomic status, and the risk factors focused on in the WHO 25 × 25 strategy are unanimously regarded as causes of premature death (Stringhini et al., 2017). Particular attention is paid to the risk factors tobacco use, alcohol abuse, salt intake, obesity, raised blood pressure, raised blood glucose, and diabetes (Kontis et al., 2014). These two general risk factors (socioeconomic status and WHO risk factors) have not been controlled in any study of the mortality of elite athletes. In a model to explain the life span of elite athletes, both factors must be considered. Since men in the general population have a riskier lifestyle, this should also apply to male athletes after the end of their career. On the other hand, elite athletes, especially women, behave in a more risk-averse manner with regard to the general risks listed above in order to not jeopardize their sporting career.

Our analysis of German Olympic participants' mortality strengthens the evidence that sports have lost their positive effect on the life span of elite athletes and that sports can be considered a risk factor. Gender and the type of sport determine the exercise workload. In case of moderate training load, positive effects on the life span should occur. For elite athletes, however, negative effects such as sports accidents, heart failure, suicide, or doping may outweigh the positive effects. Other risk factors specific to competitive sports have not been widely observed to date but cannot be ruled out. Our analysis indicates that negative effects are stronger for male elite athletes than for female athletes and increase with higher training loads.

Gender and sport-specific workload also determine the level of sporting success. The strong evidence for gender-specific influences on mortality risk suggests that gender is a moderator for the effect of athletic success on socioeconomic status. In addition, gender might also influence the effect of exercise load on both general risk factors and factors specific to the type of sport. It can also be assumed that gender directly influences the general risk factors after the end of a sporting career. This results in four groups of effect factors, which are influenced in different ways by sporting success, training load, type of sport, and gender. Compared to a life without a competitive sports career, a higher socioeconomic status and the avoidance of general risks during the sports career lead to a longer life span. In contrast, with increasing training load, sport-specific risk factors reduce the life span. It remains to be seen whether a sports career leads to a healthier lifestyle after its end than would have been the case without a sports career. There is still a lack of research in this area, so that the direction of the effect cannot be stated at present.

**Figure 3:**
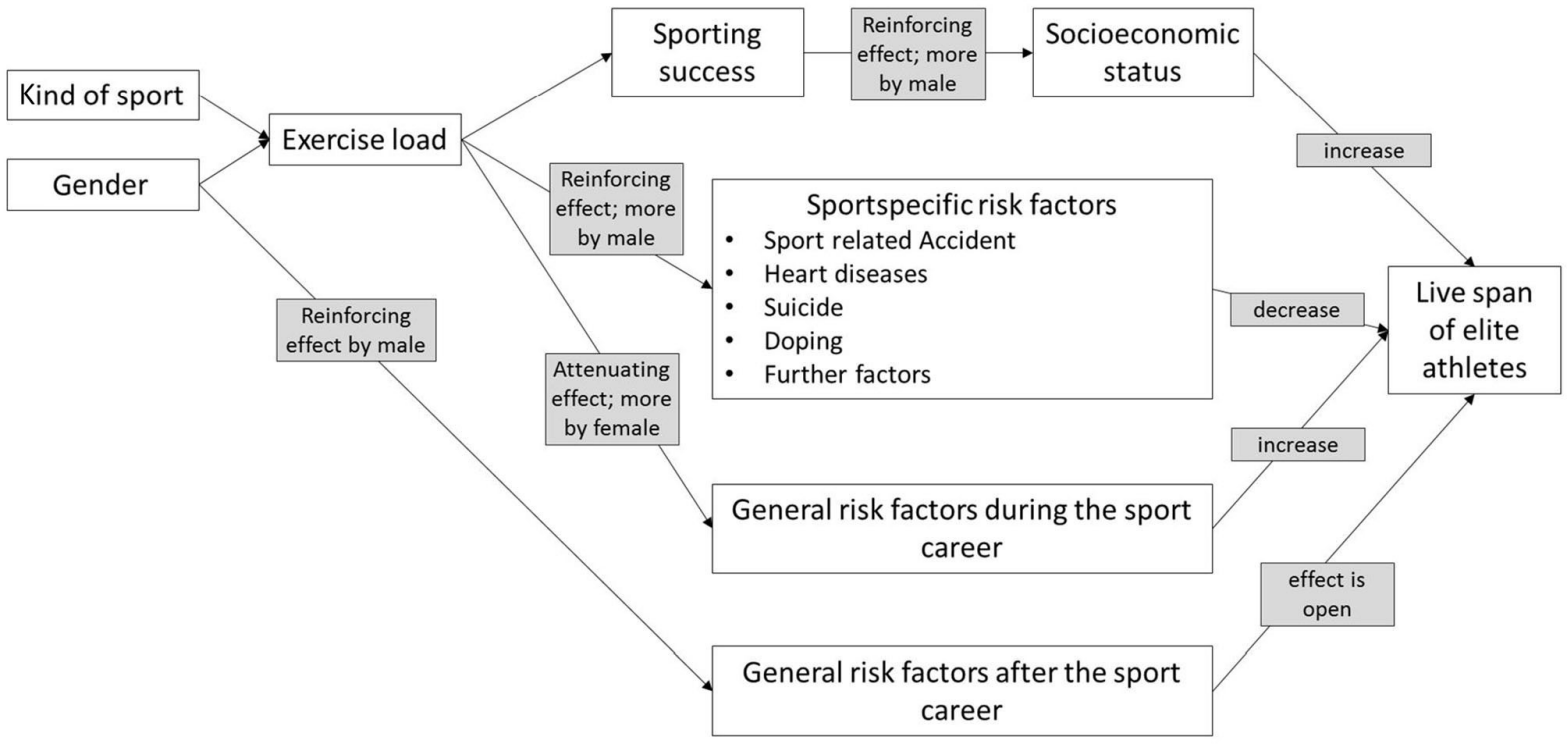
Model to explain the life span of elite athletes.

The above considerations lead to a model which aims to explain the life span of elite athletes and postulates the influencing variables and effects, which are summarized in Figure 3.

The current state of research, enriched by the results of our analysis, now allows a first proposal for a training impact model on the life span of athletes at the international top level. However, the evidence gathered has not yet reached a quality that would allow further insights into causal relationships.

In summary, the assumption of a higher survival rate of Olympians compared to the general population was not confirmed. On the contrary, it was found that Olympic success represents a linear risk for survival probability. While different types of sport do not exhibit any differences, gender and origin (FRG vs. GDR) do represent a significant risk factor. Based on our results and the current state of research, we have proposed an impact model that integrates all of the results to date but also identifies the missing impact factors. As for all survival analyses, the results presented are limited due to the usage of censored data. Furthermore, it is possible that due to data limitations, certain deaths were not taken into account in our analysis, and thus actual mortality risk for elite athletes may be even higher than currently stated. However, overall, we do not believe a systematic distortion in favor of one of the comparison categories (e.g., origin, gender, type of sport) to be likely. Also due to data limitations, only sports-specific risk factors were controlled. Data on suicide and risk factors that are usually very influential in general survival analyses, such as socioeconomic status (income, property, professional position, reputation of the profession), and educational risk factors had to be neglected. Also, it is questionable whether comparisons of official causes of death would be appropriate as they would not allow conclusions to be drawn about competitive sports-induced reasons for a risky lifestyle. Moreover, the unsolved endogeneity problem discussed above represents a limitation to the results presented. However, the proposed model could address the endogeneity problem if it withstands further validation and falsification.

The partially contradictory results call for a repeat of the analysis with a larger data set. The groups of Olympians analyzed could also be specified in more detail and extended to further countries in order to expand the reach of the results presented. In a next step, the proposed model should be tested and developed further. The results may contribute to the validity of general theories of aging.

## Data Availability Statement

The raw data supporting the conclusions of this article will be made available by the authors, without undue reservation.

## Author Contributions

All authors listed have made a substantial, direct and intellectual contribution to the work, and approved it for publication.

## Conflict of Interest

The authors declare that the research was conducted in the absence of any commercial or financial relationships that could be construed as a potential conflict of interest.
